# Advances in regenerative medicine-based approaches for skin regeneration and rejuvenation

**DOI:** 10.3389/fbioe.2025.1527854

**Published:** 2025-02-12

**Authors:** Nathalia Silva Dutra Alves, Gustavo Roncoli Reigado, Mayara Santos, Izabela Daniel Sardinha Caldeira, Henrique dos Santos Hernandes, Bruna Leticia Freitas-Marchi, Elina Zhivov, Felipe Santiago Chambergo, Viviane Abreu Nunes

**Affiliations:** ^1^ Laboratory of Skin Physiology and Tissue Bioengineering, School of Arts, Sciences and Humanities, University of São Paulo, São Paulo, Brazil; ^2^ Laboratory of Proteins and Biotechnology, School of Arts, Sciences and Humanities, University of São Paulo, São Paulo, Brazil; ^3^ Laboratory of Organic Chemistry and Molecular Biology, University of Gothenburg, Gothenburg, Sweden; ^4^ Wound Healing and Regenerative Medicine Research Program, Dr. Phillip Frost Department of Dermatology and Cutaneous Surgery, University of Miami Miller Medical School, Miami, FL, United States

**Keywords:** skin regeneration, rejuvenation, regenerative medicine, stem cells, bioengineered skin substitutes, wound healing, chronic wounds, burns

## Abstract

Significant progress has been made in regenerative medicine for skin repair and rejuvenation. This review examines core technologies including stem cell therapy, bioengineered skin substitutes, platelet-rich plasma (PRP), exosome-based therapies, and gene editing techniques like CRISPR. These methods hold promise for treating a range of conditions, from chronic wounds and burns to age-related skin changes and genetic disorders. Challenges remain in optimizing these therapies for broader accessibility and ensuring long-term safety and efficacy.

## Graphical Abstract

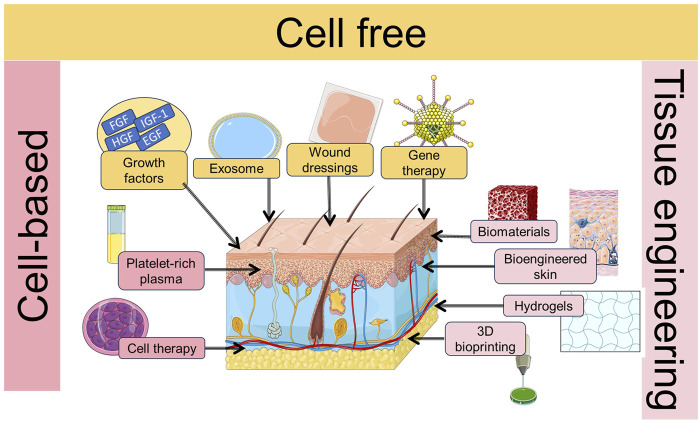

## 1 Data collection and inclusion/exclusion criteria

This review systematically analyzed articles published in English from 2015 to 2024 focusing on regenerative medicine approaches for skin regeneration and rejuvenation. Searches were conducted using PubMed, Scopus, and Web of Science databases. Keywords included combinations of “skin regeneration”, “skin rejuvenation”, “regenerative medicine”, and specific treatment modalities (e.g., “stem cell therapy”, “platelet-rich plasma”, “exosomes”). Studies were included if they presented original research or comprehensive reviews related to the specified topic. Exclusion criteria included studies not published in English, those focusing solely on animal models without human relevance, and those not meeting minimum methodological quality standards such as lack of adequate controls, small sample sizes.

## 2 Skin regeneration and rejuvenation

Regenerative medicine is a developing field focused on the repair, rejuvenation, replacement, or regeneration of tissues and organs to reestablish normal function (Mao and Mooney, 2015; Sampogna et al., 2015; Jafarzadeh et al., 2024). In the context of skin, regenerative medicine offers innovative approaches to healing (Shimizu et al., 2022; Mahajan et al., 2024) and rejuvenating the skin (Jo et al., 2021; Taub, 2024), the body’s largest organ and serves as the first line of defense.

The skin is a complex, multi-layered organ composed of the epidermis (outer layer), dermis (middle layer), and hypodermis (subcutaneous tissue). It possesses several critical functions such as a) barrier protection against pathogens, ultraviolet radiation (UV), and physical injury; b) temperature regulation, maintaining body temperature through sweat production and blood vessel dilation or constriction, c) sensory perception, since it contains nerve endings and d) immune defense, where host immune cells protect against infections and participate in wound healing (Yousef et al., 2024).

Skin can be damaged by several factors, including injury, diseases, aging, and environmental stressors (Parrado et al., 2019; Arabpour et al., 2024). Traditional treatments often focus on managing symptoms rather than focusing on the underlying damage. On the other hand, regenerative medicine for skin focuses on repairing or replacement of damaged skin tissue, restoring the skin normal function and appearance by promoting the regeneration of the tissue (Fadilah et al., 2022). Innovative techniques, including stem cell therapy, tissue engineering, and growth factors, have been developed to address conditions such as chronic wounds, burns, scars, chronic ulcers, and aged skin (Shimizu et al., 2022). These approaches aim to accelerate healing, minimize scarring, and restore skin integrity (Fadilah et al., 2022).

Specifically, chronic wounds, such as diabetic foot ulcers (DFU), venous leg (VLU) or pressure ulcers, can be difficult to treat and often fail to heal with conventional methods (Frykberg and Banks, 2015; OuYang et al., 2023). Regenerative approaches such as stem cell therapy and bioengineered skin can promote faster and effective healing in DFU (Chiu et al., 2023). Platelet-rich plasma (PRP), which is rich in growth factors, can be used to enhance healing and regeneration in chronic skin conditions where skin healing is impaired (Xu et al., 2020).

Also, severe burns can result in significant tissue loss and scarring. Traditional burn treatments often involve skin grafts, which can be painful and leave significant scarring (Chogan et al., 2023). On the other hand, regenerative medicine, requires advanced therapies like bioengineered skin grafts, stem cell treatments, exosome therapy, topical application of growth factors, such as Epidermal Growth Factor (EGF) and Platelet Derived Growth Factor (PDGF) directly to burn wounds, to promote faster and more effective healing (Chogan et al., 2023), reducing scarring, and improving functionality, elasticity, and sensitivity, as well as aesthetic outcomes. Additionally, advanced dressings and scaffolds are being proposed such as hydrogel dressings, which provide a moist environment that promotes healing and reduces pain. They can also be infused with growth factors, stem cells, or other regenerative agents to further enhance wound healing (Surowiecka et al., 2022). As far as burned skin, fractional CO_2_ laser therapy can be applied to remodel scar tissue and improve the texture and elasticity of the skin. This therapy can also be combined with stem cells or growth factors, as treatments to enhance skin regeneration (Roohaninasab et al., 2023). In case of burns, many regenerative treatments, such as stem cell therapy can also be tailored to the individual patient, reducing the risk of immune rejection and improving outcomes (Lukomskyj et al., 2022).

Chronic skin conditions like psoriasis, eczema, and vitiligo often present significant challenges in terms of management and treatment. Regenerative medicine offers new perspectives by targeting the underlying causes of these diseases and promoting long-term regeneration of skin (Paganelli et al., 2020; Daltro et al., 2020; Park et al., 2019; Bellei et al., 2022). It has been shown, for example, that mesenchymal stem cells (MSCs) can reduce the hyperproliferation of skin cells and modulate the immune response, potentially leading to remission of symptoms in autoimmune-related skin conditions like psoriasis and eczema (Shin et al., 2017; Daltro et al., 2020; Diotallevi et al., 2022). Also, exosomes can be topically applied or injected into affected areas to improve skin health and reduce the symptoms of chronic skin conditions (Wang et al., 2019; Farabi et al., 2024). In vitiligo, exosomes may help restore pigmentation by stimulating melanogenesis and melanocyte-stimulating factors (Wong et al., 2020). Gene therapy is another modality that offers a promising approach to correcting the genetic mutations underlying chronic skin diseases. In conditions like epidermolysis bullosa, a severe blistering disorder, gene therapy can be used to restore functional genes in skin cells, potentially improving skin integrity and improving skin integrity and reducing blister formation (Bischof et al., 2024). In addition, CRISPR/Cas9, a gene-editing technology has the potential to correct mutations at the DNA level, a potential solution for certain genetic skin diseases (Abdelnour et al., 2021).

Finally, skin aging, a complex interplay of internal and external factors, can now be targeted with regenerative medicine (He et al., 2023). Since skin health is considered one of the main factors associated with welfare and the perception of health in humans, numerous anti-aging strategies have been developed and proposed (Ganceviciene et al., 2012). In the context of regenerative medicine and skin rejuvenation, anti-aging therapies focus on reversing or slowing down the signs of skin aging, which includes wrinkles, loss of elasticity, pigmentation, and thinning. The aim is to repair damaged skin, promote and/or stimulate collagen production, and restore young skin features (Wong et al., 2020; Ribaudo and Gianoncelli, 2023).

Given stem cells’ remarkable potential to differentiate into various cell types, they can be used to rejuvenate tissues and organs, enhancing their regenerative capacity (Jin et al., 2023). Mesenchymal stem cells (MSCs) are not only capable of differentiating into skin cells but also are able to release growth factors and cytokines that enhance collagen synthesis and skin tissue regeneration. In this way, stem cell-based treatments are being explored for reducing wrinkles, improving skin elasticity, and treating scars (Jo et al., 2021). Also, adipose-derived stem cells (ADSCs) can be harvested from fat tissue and used in skin rejuvenation procedures, specifically ADSCs injected into areas of the face to restore volume and improve skin texture (Surowiecka and Strużyna, 2022). Exosomes from stem cells have been shown to be particularly effective in promoting healing after procedures like laser therapy or microneedling, as they can accelerate skin regeneration (Prasai et al., 2022). In skin care, topical growth factors such as EGF and transforming growth factor-beta (TGF-β) or injected formulations can stimulate collagen synthesis, accelerate wound healing, and reduce the appearance of fine lines (He et al., 2023). In the same line, studies suggest that PRP can improve fine lines, wrinkles, and overall skin texture by promoting cellular repair and enhancing skin regeneration (Phoebe et al., 2024). Lately, gene editing tools such as CRISPR are being explored to repair age-related genetic damage (Yu et al., 2022) as well as some therapies targeting genes associated with aging, like telomerase activation, have been proposed to extend the lifespan of cells (Tenchov et al., 2024). Finally, advances in 3D bioprinting and tissue scaffolds are enabling the development of engineered skin substitutes for cosmetic applications (Pleguezuelos-Beltrán et al., 2024). These skin substitutes can provide a platform for delivering stem cells or growth factors to damaged skin, promoting regeneration and rejuvenation.

Skin regenerative medicine generally encompasses two key approaches: cell-based and cell-free therapies. These include stem cell therapy, platelet-rich plasma (PRP), growth factors, cytokines, wound dressings, gene therapy, and tissue engineering, encompassing the use of biomaterials, skin grafts, bioengineered skin substitutes, and 3D bioprinting.

## 3 Cell-based therapies

### 3.1 Stem cells

Stem cells are undifferentiated cells with the ability to self-replicate, self-renewal, homing and plasticity potential. They can differentiate into various cell types, such as nerve cells, cardiomyocytes, liver cells and skin cells (Jin et al., 2023). They possess anti-inflammatory properties, promote epithelial cell proliferation, inhibit wound scarring, maintain homeostasis, repair tissue injuries, and accelerate healing (Jin et al., 2023; Khandpur et al., 2021; Zhang and Huang, 2023). Stem cells also secrete bioactive molecules, including growth factors, cytokines, chemokines and exosomes, which are responsible for the paracrine effects of these cells in bone tissue and nervous system regeneration, as well as in wound healing and endothelial cells (Tran et al., 2023; Wu et al., 2024).

There are different sources for using stem cells in regenerative medicine classified according to their differentiation potential (Khandpur et al., 2021), including embryonic stem cells (ESCs), umbilical cord mesenchymal stem cells (UCMSCs), induced pluripotent stem cells (iPSCs), MSCs, ADSCs and bone marrow mesenchymal stem cells (BMMSC) (Jin et al., 2023; Semsarzadeh and Khetarpal, 2022; Tran et al., 2023).

MSCs are originated from the mesoderm and ectoderm, and can differentiate into various cell types, such as osteocytes, chondrocytes, adipocytes, neurons and endothelial cells (Ma et al., 2023; Tran et al., 2023). Additionally, MSCs can be found in bone marrow, adipose tissue, synovial tissue, muscle, lung, liver, and umbilical cord blood (Khandpur et al., 2021; Huynh et al., 2022; Jin et al., 2023). MSCs play a pivotal role in wound healing and exhibit additional functions including immunomodulation, anti-inflammatory and anti-apoptotic effects, and pro-angiogenic activity (Khandpur et al., 2021; Wu et al., 2024).

Currently, there are several therapies using MSCs for the treatment of graft-versus-host disease, bone defects, ischemic heart failure, burns, autoimmune-related skin conditions, and diabetic foot (Jin et al., 2023; Wu et al., 2024; Farabi et al., 2024). Since MSCs can differentiate into skin cells such as keratinocytes, fibroblasts, and endothelial cells, they can be used to enhance the healing process. This is not only due to their cell differentiation capabilities but also because of their crosstalk with macrophages, which play a role in the wound repair process (Wu et al., 2024).

MSCs can be applied to burn injuries by inhibiting cellular inflammation through the release of anti-inflammatory cytokines. Additionally, MSCs exert paracrine effects that polarize macrophages from an inflammatory phenotype (M1) to a wound-healing phenotype (M2), promoting tissue repair and clearance (Ma et al., 2023; Wu et al., 2024; Zhang and Huang, 2023). Moreover, they stimulate the formation of new blood vessels, which results in increased blood perfusion and the delivery of nutrients to affected areas, thereby accelerating the healing process (Jin et al., 2023; Semsarzadeh and Khetarpal, 2022). On top of that, MSCs improve the wound structure by secreting proteins that make up the extracellular matrix (ECM), such as collagen, elastin and fibronectin, enhancing the reconstruction of the dermis (Mazini et al., 2020; Tran et al., 2023; Wu et al., 2024).

Beyond that, bone marrow-derived MSCs can be used for scars reduction, anti-aging, and systemic sclerosis (Tran et al., 2023). Conget et al. (2010) conducted a study in which they used these cells to treat two patients with severe generalized recessive dystrophic epidermolysis bullosa (EB). After 12 weeks of intradermal treatment, the ulcers healed completely, however this effect only lasted 4 months.

Another type of stem cells are the iPSCs. They are capable of differentiating into more cell types compared to MSCs, and they have the ability for unlimited self-renewal (Zhang and Huang, 2023). Studies show that these cells promote angiogenesis, perfusion, collagen deposition, and accelerate the natural healing process in murine models (Clayton et al., 2018; Farabi et al., 2024).

Besides MSCs and IPSCs, ADSCs are another promising cell type for regenerative therapy. ADSCs have shown potential applications in dermatology and aesthetics, including scar reduction, anti-aging, wrinkle reduction, and hair loss treatment (Khandpur et al., 2021; Suh et al., 2019; Tran et al., 2023). Anderi et al. (2018) injected ADSCs, derived from liposuction, into 20 patients with alopecia areata, and after 6 months of follow-up, they observed a statistically significant difference in hair growth rates among all treated patients. Indeed, Zhang et al. (2014) evaluated the antioxidant effects of ADSCs in a mouse model and found that, after 28 days of injection, ADSCs reversed the aging phenotype, increased dermal thickness and collagen content, and enhanced skin vascular density. In addition, Chen et al. (2020) showed that ADSCs were effective in improving the appearance of the skin, particularly in reducing wrinkles in UV-damaged skin. These results highlight the paracrine effects of ADSCs in promoting skin rejuvenation.

Interestingly, Li et al. (2016) used the conditioned medium from ADSCs (ADSC-CM) to evaluate its effect on hypertrophic scars *ex vivo*, injecting them into rats. Through Western blotting, they analyzed key proteins involved in healing, such as collagen I, collagen III, and α-SMA (alpha-smooth muscle actin). The results showed that the use of ADSC-CM reduced collagen deposition in hypertrophic scar tissues and improved fibrosis in these tissues.

Not only ADSC-CM, but also conditioned medium from MSCs (MSC-CM) have been investigated to be used on regenerative medicine because it contains anti-apoptotic, anti-inflammatory and anti-aging substances. Because of that, there are pre-clinical studies using MSC-CM to show whether it influence on lung progenitor cell development or its paracrine effect influences tissue regeneration (Smolinská et al., 2023).

Yet another ADSCs derivative, nano-fat, obtained through a multi-step process involving mechanical digestion and filtration of fat tissue, has demonstrated several benefits, including reduced scar size, improved skin color, and enhanced overall skin quality (Suh et al., 2019; Hajimortezayi et al., 2024).

In addition, UCMSCs can also be used in regenerative medicine, having advantages over other types of stem cells because they are abundant, easy to collect, cause no harm to donors, have low immunogenicity, and high differentiation capacity (Jin et al., 2023; Li et al., 2024). They can be used for the treatment of cardiovascular diseases, liver diseases, degenerative muscle diseases, and autoimmune diseases (Wu et al., 2024). They can also be used for treating burns and psoriasis vulgaris (Tran et al., 2023) and for treating chronic wounds, facial and body rejuvenation, even combination therapies with other biomaterials (Li et al., 2024).

The Stromal Vascular Fraction (SVF) is isolated from adipose tissue and contains various cell types, such as MSCs, endothelial cells, stromal cells, and immune cells (Surowiecka and Strużyna, 2022). For example, in a pilot study, SVF was used for the treatment of alopecia, where a significant increase in hair growth in patients was observed (Semsarzadeh and Khetarpal, 2022).

While various stem cell types exist, extensive preclinical and clinical research suggests that mesenchymal stem cells (MSCs) and MSC-based products offer the most promising balance of safety and efficacy, with a low risk of tumor formation and minimal immune rejection (Hoang et al., 2022; Farabi et al., 2024). Despite their widespread use, MSCs have limitations, such as a decline in viability and activity with age (Wu et al., 2024).

### 3.2 PRP–platelet rich plasma

PRP was first used in 1954 to improve wound healing in dentistry (Kingsley, 1954). Since its inception, the application of PRP has grown significantly, becoming a valuable tool in tissue repair and regeneration across various medical fields. (Cecerska-Heryć et al., 2022). The treatment is based on injections of the patient’s own platelets highly concentrated in plasma and separated from other blood components by centrifugation cycles (Samadi et al., 2019; Pixley et al., 2023).

The peripheral blood contains at least five times fewer platelets than PRP. This increased platelet concentration in PRP enhances its potential for wound healing (Davies and Miron, 2024). Platelets are involved in a wide range of growth factors, proteins, cytokines and other biological agents that have effects in processes like cellular migration, proliferation, differentiation, angiogenesis, tissue regeneration and collagen synthesis (Pincelli et al., 2024). The variety of molecules derived from PRP enables efficient wound healing, as the healing process involves multiple molecules and complex pathways (Samadi et al., 2019; Davies and Miron, 2024).

Platelet-derived molecules could be secreted by α-granules, dense granules and lysosomes. Platelet derived growth factor (PDGF), insulin-like growth factor 1 (IGF-1), EGF, TGF-β, fibroblast growth factor (FGF) and vascular endothelial growth factor (VEGF) are secreted from α-granules, in addition to adhesive proteins, coagulation factors, angiogenic regulators, cytokines and exosomes. They are the more abundant secretory granules and are responsible for releasing the greatest number of molecules with a direct effect on wound healing (Cecerska-Heryć et al., 2022; Pincelli et al., 2024). On the other hand, dense granules contribute to platelet activation and subsequent release of α-granules constituents, in which it has been shown that PRP lysosomes functions participate in antimicrobial activity and the degradation of extracellular matrices (Everts et al., 2024).

The composition of PRP can vary depending on the preparation. It can be categorized as pure (P-PRP), leukocyte-poor (LP-PRP), leukocyte-rich (LR-PRP) or platelet-rich fibrin (PRF), considering the centrifugation time, speed and the presence or absence of non-autologous anticoagulants (Karimi and Rockwall, 2019; Everts et al., 2024). PRF specifically has gained prominence in regenerative medicine in recent years due to its benefits, such as the release of platelet-related therapeutic granules over a longer period and at a slower rate than PRP (Narayanaswamy et al., 2023). In this way, PRF also offers a valuable adjunct to both surgical and non-surgical interventions, demonstrating great potential for enhancing treatment outcomes.

PRP treatment offers several advantages, including low cost, ease of preparation, versatility, and safety. However, further research is necessary to standardize preparation procedures and establish regulations regarding the composition of PRP injectables. Additionally, the efficacy of PRP and its various categories remains uncertain in relation to specific diseases and clinical conditions (Everts et al., 2020; Cecerska-Heryć et al., 2022; Pincelli et al., 2024).

## 4 Cell-free therapies

### 4.1 Exosomes for skin wound healing and rejuvenation

Micro-vesicles, currently known as exosomes, were first identified in 1983. Initially, exosomes were considered just a cell waste, however, later research revealed that exosomes play important roles in cell communication and signal transduction. As an alternative for cell-based therapies, exosomes emerged as potential tools for treating skin’s conditions, such as improving wound healing and even skin rejuvenation. Since exosomes are small vesicles secreted by different cells, there are many possibilities for their isolation and use as an effective carrier for bioactive compounds or genetic material. Exosomes not only transport and protect molecules from degradation, but also exhibit biocompatibility, reducing the risk of immune reactions and tumorigenesis. In this way, clinical application of exosomes is a promising avenue for free-cell therapies (Hajialiasgary Najafabadi et al., 2024; Peña and Martin, 2024; Quan, 2023; Sonbhadra and Pandey, 2023; Zhou et al., 2023).

Exosomes are extracellular vesicles, approximately 30–200 nm in size, which can be obtained from cell culture and that function as membrane-bound carriers of biomolecules and metabolites, reflecting their cellular origin. These vesicles, spherical in solution, exhibit a lipid bilayer structure, which decreases the risks of immune responses and provides protection of their cargo from degradation. Furthermore, exosomes pose a low risk of uncontrolled cell proliferation and differentiation, minimizing concerns related to tumorigenicity. Their ability to carry and deliver bioactive substances to cells makes exosome-based therapies a promising possibility for therapeutic applications like various skin conditions, including psoriasis, atopic dermatitis and vitiligo, as well as for promoting skin regeneration, such as in diabetic wound healing, hypertrophic scarring and keloid formation, and even for addressing skin aging (Gurung et al., 2021; Hajialiasgary Najafabadi et al., 2024; Yu et al., 2024).

Exosomes are ubiquitous in various body fluids, including serum, saliva, milk, cerebrospinal fluid, urine and semen. Among these, stem cell-derived exosomes have been extensively investigated for their potential role in mediating the biological effects of paracrine factors, particularly in wound healing (Zhou et al., 2023).

Tissue repair is a complex process involving initial clot formation, followed by inflammatory cell signaling, cell proliferation and remodeling. These phases overlap, each with its own purpose and time before the next starts. Mesenchymal stem cells exosomes (MSC-exos) derived from different tissues, like ADSC, hold a promise for cutaneous repair due to their ability to stimulate fibroblast activity. In the dermis, fibroblasts play a pivotal role in wound healing through collagen synthesis, making MSC-exos, which enhance fibroblast function, valuable contributors to the healing microenvironment and the promotion of wound repair. ADSC-derived exosomes demonstrate significant potential for treating diabetic wounds due to their ability to induce collagen I and III production in the early stages of healing, which can help prevent scar and keloid formation (Song et al., 2023; Zhou et al., 2023; Peña and Martin, 2024).

Data from an ECM loaded with ADSC-exosomes indicate that this combination is also a possible therapeutic approach for both normal and pathological wound healing. The results demonstrated that ECM-loaded exosomes promoted increased cell growth, cell migration, collagen deposition, and decreased inflammation *in vivo* (Song et al., 2023).

Moreover, given their role in wound healing, stem cell-derived exosomes could potentially be used to treat skin photoaging, a condition marked by uneven pigmentation and wrinkles (Hajialiasgary Najafabadi et al., 2024).

Park et al. (2023) demonstrated that exosomes derived from human foreskin fibroblasts (BJ-5ta Exo) can mitigate oxidative stress by upregulating the expression of antioxidant genes CAT, SOD-1, SOD-2, and GPX. Additionally, BJ-5ta Exo promoted a decrease in programmed cell death and cell cycle arrest.

In addition, it was suggested that exosomes obtained from 3D culture of human dermal fibroblasts (HDFs) may be able to promote collagen synthesis and reduce skin inflammation. Data also revealed that 3D-HDF-exos, through tumor necrosis factor alpha (TNF-α) downregulation and TGF-β upregulation, promoted a procollagen type I increase while reducing matrix metalloproteinase-1 (MMP-1) expression. Given the age-related decline in HDF collagen production and repair, coupled with increased MMP-mediated ECM degradation, these findings suggest that exosomes may possess anti-aging properties (Xiong et al., 2021).

Given the abundance of exosomes in bovine milk, recent studies have explored the potential of milk-derived exosomes (MK-exo) as novel anti-aging compounds. These investigations have revealed that MK-exo can stimulate the expression of filaggrin and CD44 in keratinocytes, as well as hyaluronidase levels in fibroblasts. Furthermore, MK-exo protected collagen biosynthesis from UV-induced damage. Notably, these exosomes also stimulated increased cell migration rates in fibroblasts (Lu et al., 2024).

However, while exosomes demonstrate significant promise as therapeutic agents, several challenges must be addressed for their clinical application. These include standardizing isolation and analysis, ensuring safety and purity, and preserving exosome activity during preparation and storage (Lu et al., 2024; Rezaie et al., 2022).

Despite above stated challenges, exosomes, being versatile carriers of biomolecules, offer promise for clinical applications. Their biocompatibility, low risk of immune responses and tumorigenicity make them attractive candidates for treating diseases. In the context of skin, their potential to influence collagen synthesis and inflammation suggests their value for wound healing and skin rejuvenation. Future research should focus on exploring these possibilities and ensuring the safety of exosomes for medical use.

### 4.2 Pharmaceuticals, growth factors, fillers, gene therapy

Dermal fillers, also known as facial fillers, are soft, gel-like substances injected beneath the skin. Over time, the loss of soft tissue volume, fat redistribution, reduced skin elasticity, and thickness contribute to the formation of wrinkles and folds characteristic of aging. In this context, injectable fillers are an option for treating wrinkles, scars, folds, and areas under the skin lacking volume (Callan et al., 2013; Maio, 2018; Colon et al., 2023; Faris, 2024).

The principle behind the use of these products is based on strengthening the ECM in the dermal layer. They are made of various low-crosslinking polymeric ingredients with different effects on the skin (Yi et al., 2024). Among the most used ingredients are hyaluronic acid, poly-L-lactic acid, polymethylmethacrylate, and calcium hydroxyapatite (CaHA) (Colon et al., 2023).

Hyaluronic acid is the most commonly used dermal filler, naturally found in the skin, being safe and effective for use (Yi et al., 2024). Poly-L-lactic acid is synthetic, acting as a collagen stimulator, recommended for softening lines and treating wrinkles (Ao et al., 2024). Similarly, according to the American Board of Cosmetic Surgery (2022), polymethylmethacrylate comprises synthetic microspheres that remain under the skin indefinitely, providing continuous support and containing collagen in their composition. In addition, CaHA is a natural substance found in bones that helps stimulate the skin’s natural collagen production and is recommended for treating deeper lines.

Focusing on hyaluronic acid fillers due to their widespread use, it is the most abundant glycosaminoglycan found in the human dermis, contributing to tissue hydration and volume, as well as providing structural support (Ballin et al., 2015; Wongprasert et al., 2022). A recent study by Chen et al. (2023) demonstrated the anti-aging ability of hyaluronic acid fillers by inhibiting the expression of MMP-1, promoting collagen accumulation, and increasing the expression of dermo-epidermal junction proteins.

One of the most significant advantages of hyaluronic acid fillers is that they can be easily removed by injecting hyaluronidase (Wongprasert et al., 2022). However, some limitations persist in their use, which can sometimes extend to other types of fillers, such as the duration of the compounds, discomfort caused by the injections, and the ability to achieve precise delivery of the fillers to the intended location and skin layer (Colon et al., 2023).

Growth factors are polypeptides or proteins that regulate physiological processes in and between cells. They are naturally found in the skin, secreted by various cell types in this tissue. Many growth factors are involved in wound healing, both acute and chronic, and are some of the most important signalers during this process (Pamela, 2018; Yamakawa and Hayashida, 2019; Vaidyanathan, 2021). Furthermore, the processes of aging and wound healing naturally stimulate the release of growth factors, which affect critical biochemical repair pathways in the dermal matrix and have inspired the use of these factors to improve skin appearance (Kremer and Burkemper, 2024).

Some of the growth factors used in skin treatment are EGF, FGF, hepatocyte growth factor (HGF), and IGF-1, which can be used in combination (Vaidyanathan, 2021; Yamakawa and Hayashida, 2019). They can come from various sources, including human and non-human cell cultures and recombinant sources (Quinlan et al., 2023).

Treatments involving the use of different growth factors have been reported to improve wrinkles, skin texture, photo-damage, and the overall appearance of facial skin (Quinlan et al., 2023). In addition to cosmetic use, human EGF (hEGF) is also applied in regenerative medicine for the treatment of alopecia, dermatitis following chemotherapy, burns, diabetic foot ulcers, and post-surgical ulcers (Kong and Hong, 2013; Kim et al., 2017; Esquirol-Caussa and Herrero-Vila, 2019; Jeon et al., 2019; Kahraman et al., 2019; Lou, 2021; Vaidyanathan, 2021).

The use of EGF for aesthetic purposes also shows broad applications, presenting a regenerative effect in the aging skin process by promoting the migration of aged fibroblasts and increasing the synthesis of hyaluronic acid and collagen in this tissue (Kim et al., 2015; Miller-Kobisher et al., 2021; Vaidyanathan, 2021). Growth factors can also be combined with other treatments for apparent skin rejuvenation, such as lasers, microneedling, radiofrequency, or chemical peels, to amplify results or improve side effects from these treatments (Quinlan et al., 2023). However, in recent years, the development of cell-penetrating peptides associated with growth factors has improved the topical application of these factors, increasing their ability to penetrate the skin and epithelial cells (Chen et al., 2017; Choi et al., 2018; Jeon et al., 2019, Lee et al., 2020, Xie et al., 2020.

Aiming to correct genetic defects and thereby prevent or cure genetic disorders, gene therapy has been applied to skin regenerative medicine. All types of skin diseases are candidates for gene therapy, from inflammatory diseases to skin cancers and genodermatoses (Ain et al., 2021), as well as more aesthetic aspects like skin regeneration and scar and keloid treatment (Hosseinkhani et al., 2023; Luo et al., 2023).

Gene therapy technologies include the use of messenger RNA, silencing RNA, antisense oligonucleotides (AON), plasmid DNA, minicircle DNA, mini-string DNA, and CRISPR/Cas9 technology (Wan et al., 2021; Guri-Lamce et al., 2024; Tenchov et al., 2024), which must have specific action on the desired area of the skin, representing one of the current challenges in this type of therapy.

Currently, there are three main methods of delivering gene therapies to the skin: viral delivery, nanoparticles and physical methods. In this perspective, viral delivery is the most used and effective method (Picanço-Castro et al., 2020), though it presents challenges such as limitations in efficacy due to pre-existing immunity, the inability to redose, and genome integration capacity, which increases the risk of unwanted insertional mutagenesis. Additionally, there are size limitations for the genetic cargo (Anzalone et al., 2019; Ain et al., 2021). Lipid-based nanoparticles, a more recent and promising method (Guri-Lamce et al., 2024); polymeric nanoparticles, capable of associating with negatively charged genetic cargoes and forming spherical complexes, but which still present high toxicity related to particle size (Blakney et al., 2020); and physical methods, such as electroporation, ultrasound application, or microneedles (Wan et al., 2021), which have limitations for clinical application, such as limited cargo capacity and challenges with whole-body administration (Ain et al., 2021).

Other substances can be incorporated into skin care products, playing an active role in tissue regeneration or inflammation by improving ECM synthesis or inhibiting its degradation, neutralizing free reactive species, or reducing proinflammatory factors (Makpol et al., 2013; Wang H. et al., 2021; Torres et al., 2023). Among these substances, zinc-based compounds stand out. This metal has been shown to reduce inflammation and the risk of infections, being involved in cell proliferation and migration and collagen synthesis, thus promoting epithelialization (Lin et al., 2017; Chen et al., 2022; Pino et al., 2023). Other substances primarily act as moisturizers, provided by ingredients that increase the synthesis of structural skin lipids, directly restoring the skin barrier, such as vitamin A, or hygroscopic substances like dexpanthenol, which bind to and retain water in the stratum corneum (Liu, 2022). Other substances are lipids, oils, and fatty acids, known as emollients, such as petrolatum derivatives, which also promote hydration by directly replacing missing fatty acids in the tissue (Elias, 2022; Torres et al., 2023).

### 4.3 Wound dressings

Smart dressings were developed to enhance wound care management based on the injury type and patient conditions (Raju et al., 2022). Multifunctional wound dressings promote wound healing by encapsulating bioactive substances, sustaining the release of medicines by stimuli-responsive technology (Raju et al., 2022). Biopolymers such as alginate, chitosan, polyvinyl alcohol (PVA), and collagen are increasingly used to create innovative wound dressings due to their cost-effectiveness and eco-friendliness. Among these, alginate dressings are the most popular because they promote skin regeneration, accelerate wound closure, minimize scarring, absorb exudates effectively, and are biocompatible. Alginate’s gelling properties and stability in warm environments make it ideal for various applications, including hydrogels, nanofibers, dermal patches, films, and foams (Nqoro et al., 2022).

Other than that, hydrocolloids are effective in wound care because they can absorb significant amounts of wound fluid and are impermeable to water vapor, creating a moist healing environment. They also block oxygen, which speeds up epithelialization and collagen synthesis while lowering the pH to reduce bacterial growth (Nguyen et al., 2023). Nonetheless, new smart hydrogel wound dressings with embedded sensors have been rapidly developed to monitor wound conditions. Notable examples include flexible pH-sensing alginate-based hydrogel fibers for skin wounds and PVA/xyloglucan (PVA/XG) hydrogel membranes that absorb exudate and release biological factors (Tamayol et al., 2016; Ajovalasit et al., 2018; Tavakoli and Klar, 2020).

Although high-end wound dressings have been developed in recent years, the products face several limitations, including a complicated production process, inadequate quality assurance for biological materials and questions about the effectiveness of their components for widespread use. In addition to that, more trials and experiments are needed to assess the true effectiveness of these advanced dressings in wound healing (Nguyen et al., 2023).

## 5 Skin bioengineering

Tissue engineering has become a multidisciplinary research field that involves the use of techniques to replicate biological prototypes, such as skin, to study the regeneration of physiological tissue on repairing or replacement of damaged skin (Berthiaume et al., 2011; Deepa and Bhatt, 2024; Wei et al., 2024). Indeed, it is possible to introduce biomaterials and hydrogels, which can be used as scaffolds to facilitate wound healing, combined with the knowledge of cell culture, for the improvement of techniques such as the use of nanomaterials and 3D bioprinting (Kondej et al., 2024; Wei et al., 2024; Loukelis et al., 2024; Bian et al., 2024).

### 5.1 Organotypic cultures – 3D *in vitro* skin

Studies in the last 20 years have involved the use of different skin cell cultures (mainly keratinocytes), umbilical cord mesenchymal stem cells differentiated to keratinocytes, or co-culture of skin cells with other cell types, including immune cells and dermal fibroblasts, in a two-dimensional (2D) monolayer (Abaci et al., 2017; Santos et al., 2023), to study the signaling pathways of skin diseases such as psoriasis or melanoma, wound healing, also to test the efficacy of safety treatments (Karras and Kunz, 2024). However, 2D models do not often represent a sufficient level of complexity to assess the various cell-cell and cell-extracellular matrix interactions, as well as oxygen and nutrient gradients (Loke et al., 2021; Santos et al., 2023).

On the other hand, it is possible to better understand most complex skin diseases in the three-dimensional (3D) microenvironment by involving additional cell lineages, such as immune cells or skin appendages as innervation type, to generate more effective *in vitro* skin models, using spheroids or skin constructs, for example, to improve skin replacement therapy (Abaci et al., 2017; Karras and Kunz, 2024; Loke et al., 2021).

### 5.2 Spheroids

The *in vivo* model can be better mimicked in spheroid cultures compared to 2D models, representing a more complex tissue architecture, with increased cell-cell contacts and heterogeneous cell growth. In addition, spheroids have been used for semi-high-throughput drug screening, as well as being used in co-culture models to evaluate different tissue responses under paracrine stimulation (Raghavan et al., 2016; Loke et al., 2021; Karras and Kunz, 2024).

The application of spheroid cultures in skin models can be generated from cell aggregates of fibroblast or keratinocyte lineages, for example, under non-adherent conditions, by so-called spinner culture, hanging drop, magnetic levitation or gel incorporation (Abaci et al., 2017; Klicks et al., 2019; Schäfer et al., 2021; Ohguro et al., 2024). To improve the formation of the spheroids, it is important to choose an ECM based on biomaterials, such as hydrogels or collagens, for the architecture of the dermal matrix (Enyedi et al., 2023; Santos et al., 2023).

Some methods for evaluating the different cell configurations in spheroids still remain limited, but the main ones based on microscopy, such as phase contrast microscopy, are used to analyze the size and shape of spheroids. Other methods, such as cell surface staining and flow cytometry, are used to analyze the presence of specific molecules. Cryosectioning, after fixation in formalin and embedding in paraffin, is used for a deeper view of the sectioned spheroid (Filipiak-Duliban et al., 2022; Habanjar et al., 2021; Karras and Kunz, 2024).

Due to the cell aggregate structure, spheroid nuclei are exposed to low oxygen conditions, as well as limited access to nutrients and metabolites, which can lead to an increase in apoptotic cells (Karras and Kunz, 2024; Loke et al., 2021). On the other hand, the cells on the periphery are proliferative, due to the availability of oxygen and nutrients. Interestingly, the middle layer contains quiescent and senescent cells, and as a result, this spheroid configuration becomes a suitable model for testing pathophysiological conditions (Karras and Kunz, 2024; Loke et al., 2021; Ohguro et al., 2023).

### 5.3 Skin reconstructs

Another model of organotypic cultures are the so-called “raft” cultures, also known as skin reconstructs. Cells are established in a manner as to allow the stratified epithelium and the dermal component of the skin, to be reconstituted in a tissue culture environment. Keratinocytes, for example, are seeded in a dermal equivalent containing fibroblast and, when raised to the air-liquid interface, reproduce the process of stratification and terminal differentiation of keratinocyte (Klicks et al., 2019; Santos et al., 2023). Histological analysis of these skin reconstructs shows the similarity and tissue organization to human skin, with a cornified epidermal-equivalent appearing on top of a dermal, containing human fibroblasts (Klicks et al., 2019; Loke et al., 2021; Santos et al., 2023).

This skin reconstruct is a useful system for testing pharmacological dynamics, efficacy tests, analysis of absorption by different forms of administration, or for preclinical screening of drugs and cosmetics (Torre et al., 2020; Portugal-Cohen et al., 2023; Suthar et al., 2024). This model takes less time to obtain results and is less expensive than performing experiments using animals (Abaci et al., 2017; Karras and Kunz, 2024). Therefore, it has been emphasized in recent years that organotypic cultures for skin reconstructions can also be obtained using the bioprinting technique, in order to construct highly reproducible dermal equivalents, with architecture similar to the *in vivo* (Cubo et al., 2016; Fernandes et al., 2022; Bian et al., 2024), to be widely used in regenerative medicine or in strategies for testing immunotherapy (Ao et al., 2022; Santos et al., 2023).

### 5.4 Bioprinting

Tissue bioengineering has been expanding as a new strategy by employing advanced techniques of bioprinting, biopolymer engineering, stem cell research and nanomedicine (Augustine, 2018; Pasierb et al., 2022; Wei et al., 2024). Bioprinting has attracted attention as a promising technique, in which the technology aims to generate, precisely, a controlled and organized complex with similar architectures of native tissues (Loukelis et al., 2024; Bian et al., 2024). Bioprinting has been used to generate tissues and transplants, including skin and its multilayers, tracheal splints, cardiac tissue and cartilaginous textures (Kaur et al., 2019; Miguel et al., 2019; Bian et al., 2024).

In fact, bioprinting technique has the potential to revolutionize contemporary regenerative medicine, considering that by taking advantage of tissue regeneration techniques. Using approaches that facilitates the production of skin and consequently its use in cases of wound closure, it is also possible within this model to mimic characteristic inflammatory profiles, in order to study drug-related toxicity or investigate the pathological mechanism of some skin diseases, including psoriasis and atopic dermatitis (Randall et al., 2018; Lorthois et al., 2019; Derr et al., 2019; Liu et al., 2020; Deepa and Bhatt, 2024).

The simultaneous incorporation of different cells into the bioprint, including fibroblasts and melanocytes in dermal equivalents, makes it possible to study the impact of UV radiation (Pasierb et al., 2022). Compared to machining prototypes, bioprinting makes skin production cheaper and faster, as the prototype can be finished in hours, allowing the process to be efficient, even with design modifications in production. The manufacturing process can also reduce material costs, as it uses only the amount of material needed for the prototype itself, minimizing or eliminating waste (Wang H. et al., 2021; Wang Z. et al., 2021).

Other advantages of using the bioprinting technique include: 1) customization of the skin to be used. Depending on the shape and depth of the wound surface, imaging technology using computer digitization can quickly print the skin graft compatible with the wound. Indeed, this technique confers the characteristics of punctuality, high flow and high repeatability (Weng et al., 2021). 2) the use of bioinks, which can be deposited flexibly and precisely with different biological agents, including living cells, nucleic acids, growth factors, among others, is usually required to help build skin structures (Zhu et al., 2016; Wei et al., 2024; Loukelis et al., 2024). According to the different printing materials, three different techniques of bioprinting can be mentioned, such as: droplet-based bioprinting (DBB), laser-assisted bioprinting (LAB) and extrusion-based bioprinting (EBB) (Gudapati et al., 2016; Weng et al., 2021; Kang et al., 2022).

### 5.5 Droplet based bioprinting (DBB)

DBB technique includes the drop-by-drop mode. In this model, drops of biomaterial on a substrate are deposited when necessary. DBB-based bioprinters are suitable for deposition and patterning of materials, due to their high precision and minimal biomaterial waste. In addition, DBB mainly uses piezoelectric, thermal or electrostatic forces to generate droplets, which can precisely deposit the biomaterial to make a spatially heterogeneous tissue structure (Gudapati et al., 2016; Matai et al., 2020; Kang et al., 2022). Its non-contact printing mode is more suitable for biological printing directly onto a wound, for example,. Some studies have used the DBB technique to print human keratinocytes and fibroblasts directly onto dermal wounds on the backs of mice. Compared to the control group without any biological dressing, the skin grafts in the experimental group promoted wound healing (Gudapati et al., 2016; Matai et al., 2020; Wang Z. et al., 2021). On the other hand, DBB has some limitations. Its inkjet injector is small, measuring up to 150 μm, which can be easily blocked by biomaterials. Only low-viscosity hydrogels or other low-concentration biological agents can be used (Wang H. et al., 2021).

### 5.6 Laser-assisted bioprinting (LAB)

LAB technique consists of the emission of laser light, that is focused on the metal film on the back of the silicate glass and heated locally, so that the bioink deposited on the equipment evaporates and is sprayed onto the substrate in the form of liquid drops (Matai et al., 2020; Wang Z. et al., 2021). The LAB technique mainly uses a nanosecond laser with ultraviolet wavelengths as an energy source, and its printing resolution can reach the picogram level, performing bioprinting without direct contact with the substrate and can print cells with high resolution. (Zhang et al., 2023). However, LAB does not have a suitable rapid gelling mechanism yet, which limits its ability to produce high-performance prints (Wang H. et al., 2021; Zhang et al., 2023).

### 5.7 Extrusion-based bioprinting (EBB)

EBB technique makes controllable impressions using fluid distribution systems and automated machines. Under the control of a computer, the biomaterial passes through a catheter, using pneumatic, piston or screw approaches (Zhang et al., 2023). Hydrogels better perform in bioprinting by pneumatic extrusion, because it is kept in this material the profile of printed filaments, after extrusion. The screw-driven structure can bioprint biomaterial at high viscosity, which is conducive to producing a more stable bioprinted tissue (Zieliński et al., 2023). In addition, extrusion bioprinting can print a porous grid structure, promote the circulation of nutrients and metabolites, which allows better control over porosity, shape and distribution of cells in the printed prototype (Pasierb et al., 2022; Zhang et al., 2023). Compared to DBB and LAB models, the advantages of EBB include faster bioprinting speed, more usable bioink types (including cell clusters, high-viscosity hydrogels, microcarriers and cell matrix components) (Pasierb et al., 2022; Zhang et al., 2023), more versatility and suitability for manufacturing prosthetic implants for tissue bioengineering. However, the limitation of this technique is that it has a lower resolution of at least 100 µm (Zhang et al., 2023; Zieliński et al., 2023).

In general, different approaches in bioprinting techniques are used, in order to allow specialists to acquire more precision and high resolution in the regeneration of the skin and its appendages, including hair follicles, sebaceous and sweat glands. The same approaches might be used on selecting between the different biomaterials, which make the skin cell lineages remain highly viable and metabolically active, to keep the accuracy in replicating the tissue layers and to not compromise functionality (Weng et al., 2021).

### 5.8 Biomaterials

Biomaterials can be of natural, synthetic, or a combination of both origins and are of great interest in tissue engineering due to their properties of biocompatibility, biodegradability, promoting cell adhesion and migration in scaffolds, potential resemblance the extracellular matrix, and presenting controllable properties and architecture (Chaudhari et al., 2016; Liu et al., 2023).

Natural biomaterials are primarily derived from proteins, such as collagen and spider silk, for example, (Liu et al., 2023), but can also originate from carbohydrates, such as alginate (Farshidfar et al., 2023).

Firstly, collagen is a protein that contains triple helices capable of forming strong and stable fibers through cross-linking. This stability can be used to form scaffolds that resemble the ECM of living tissues (Chattopadhyay and Raines, 2014; Amirrah et al., 2022; Zhu et al., 2022). Thus, in regenerative medicine, collagen as a biomaterial can be used as a wound dressing, promoting healing, or as a supplement for the skin, improving aspects such as elasticity and hydration (Ghomi et al., 2021).

Spider silk, as a natural biomaterial, is explored for its biocompatibility and low density. Because it is difficult to cultivate directly from arachnids, this protein has been produced recombinantly for the construction of scaffolds for cell culture. Using these supports, the regeneration of bone, cartilage, muscle, nerve, and epidermal tissues, especially in burn patients, has been studied. (Salehi et al., 2020).

It is important to highlight that some materials have limited properties, such as alginate, which has low stability due to its chemical properties, and polydopamine, which has low hydrogel-forming capacity. However, they can be used in combination with other materials, such as hydroxyapatite, chitosan, gelatin, and collagen, to achieve results and applications in tissue engineering. Thus, in combination, they can be used for bone tissue repair, corneal reconstruction, wound healing and covering, and even in drug delivery systems (Farshidfar et al., 2023; Yazdi et al., 2022).

Another way to apply the biomaterials, in general, is using as hydrogels, which provide a moist environment with the ability to retain proteins, growth factors, and nutrients within the gel structure and release these molecules into the medium (Berthiaume et al., 2011; Lei et al., 2022). Due to technological advancements in tissue engineering, it has become possible to work on an increasingly smaller scale of these gels, creating nanogels which can reach smaller and more internal wounds than hydrogels, ensuring drug release in the region, facilitating wound healing and tissue regeneration (Grimaudo et al., 2019; Brianna et al., 2024). In addition to hydrogels, there are also nanomaterials, which can be made of a single chemical element, such as silver or gold, that possess antimicrobial characteristics, can stimulate cell growth and can be delivered in systems along with nanogels (Bellu et al., 2021). The field of nanostructures in regenerative medicine and tissue engineering is still a novelty and shows extreme promise with ongoing research advancements.

## 6 Limitations of regenerative medicine for skin

As mentioned previously, the field of regenerative medicine for skin regeneration and rejuvenation, while promising, faces several significant limitations as described:

1. High costs and limited accessibility:many regenerative therapies, especially those involving stem cells or bioengineered tissues, are expensive and require specialized equipment and expertise, limiting their accessibility to many patients.
2. Variability in treatment outcomes: the effectiveness of regenerative therapies can vary significantly depending on factors like disease severity, individual genetic background, and overall health. This makes it challenging to predict outcomes and standardize treatment protocols.
3. Long development times and regulatory hurdles: developing and gaining regulatory approval for new regenerative therapies is time-consuming and costly, which slows down the introduction of promising treatments to the market.
4. Technical challenges in production and application: producing sufficient quantities of high-quality regenerative materials (stem cells, bioengineered tissues, exosomes) consistently and reliably for clinical applications remains a significant technological hurdle. The delivery and distribution of these materials in the body can also be complex and may not always be effective.
5. Uncertain long-term efficacy and safety: while many therapies show promise in short-term studies, the long-term efficacy and safety of regenerative treatments are often not fully established. Potential risks such as tumorigenicity or immune responses need further investigation.
6. Ethical considerations: the use of stem cells and gene editing technologies raises ethical concerns, including issues related to stem cell sources (embryonic vs. adult), the potential for off-target effects in gene editing, and the ethical considerations surrounding the use of human tissue and data. Animal testing used in preclinical research may also raise ethical questions.
7. Standardization and quality control: there is a lack of standardization in the preparation and quality control of several regenerative materials and treatments (e.g., PRP, exosomes). This affects the reproducibility and reliability of treatment outcomes.
8. Complex biological systems: the skin is a complex organ with multiple interacting cell types and signaling pathways. Fully understanding these complexities is crucial for developing truly effective regenerative therapies. Current therapies often target only a subset of these mechanisms, limiting their overall impact.
9. Limited understanding of underlying mechanisms: while many regenerative therapies show promise, a complete understanding of their precise mechanisms of action is often lacking. This limits the ability to further improve and refine treatments.

Table 1 summarizes various techniques and approaches used in skin regeneration and treatment, categorized into *in vitro* and *in vivo* methods, as well as clinical applications. For each technique, it details specific findings, strengths, and limitations or challenges encountered. The *in vitro* methods include organotypic cultures (2D and 3D), spheroids, skin reconstructs, cell cultures, exosome studies, and ADSC injections. *In vivo* techniques cover PRP injections, growth factor application, exosome application, bioprinting, animal models, stem cell and gene therapy. Finally, clinical applications include PRP therapy, growth factor therapy, and dermal fillers. The table provides a comprehensive overview of the current state of skin regeneration research and its therapeutic potential, highlighting both the advantages and drawbacks of different methodologies.

**TABLE 1:** *In vitro, in vivo* and clinical approaches to skin repair: strengths, limitations, and specific findings.

Type	Technique/Approach	Specific findings	Strengths	Limitations/Challenges
*In vitro*	Organotypic cultures (2D and 3D)	Studied signaling pathways, skin disease models (psoriasis, melanoma), and wound healing; 3D models offer greater complexity than 2D	Allows for controlled conditions and high-throughput screening; 3D models better mimic *in vivo* conditions	2D models lack complexity; 3D models have challenges in nutrient/oxygen delivery, leading to cell death in the core
	Spheroids	Used for semi-high-throughput drug screening and co-culture models; increased cell-cell contact and heterogeneous growth	Better mimicry of *in vivo* conditions than 2D	Limited access to nutrients and oxygen in the core of spheroids can affect results
	Skin reconstructs	Mimic stratified epithelium and dermal components; Useful for testing pharmacodynamics, efficacy, and absorption	Closer to *in vivo* conditions; Less time and cost than animal models	Not perfectly representative of all *in vivo* complexities
	Cell cultures (various types)	Examined differentiation potential, anti-inflammatory properties, and paracrine effects of various stem cells (MSCs, ADSCs, UCMSCs, iPSCs)	Understanding fundamental cellular mechanisms	Not directly translatable to *in vivo* outcomes
	Exosome studies	Investigated exosomes role in cell communication, wound healing, and skin rejuvenation; assessed effects of exosomes from various sources (ADSCs, HDFs, bovine milk)	Understanding mechanisms of exosome action	Challenges in isolation, standardization, and ensuring purity and long-term stability
*In vivo*	ADSCs injection	ADSCs injection into patients with alopecia areata showed improved hair growth; ADSCs reversed the aging phenotype in a mouse model	Demonstrates potential therapeutic efficacy	Limited human studies. Translation to human applications needs more research
	PRP injections	PRP injections enhanced healing in chronic skin conditions	Demonstrates potential therapeutic efficacy	Variable results depending on preparation methods; need for standardized procedures
	Growth factor application	Topical application improved wrinkles, skin texture, and photodamage; treatment of various skin conditions (alopecia, burns)	Demonstrates therapeutic potential	Effects can be localized and short-lived; may need combined therapies
	Exosome application	Topical or injected exosomes improved skin health and reduced symptoms in vitiligo and other skin conditions	Demonstrates therapeutic potential	Challenges in delivery and distribution
	Bioprinting	Generated skin tissues and transplants	Potential for creating personalized treatments	Challenges in precision, cell viability, and cost
	Animal models (various)	Used to test various approaches (stem cell therapy, exosomes, biomaterials)	Provides relevant data for assessing safety and efficacy	Results may not directly translate to human outcomes
Clinical	Stem cell therapy	Treated patients with severe generalized recessive dystrophic epidermolysis bullosa, alopecia areata, and other conditions	Demonstrates therapeutic efficacy in some cases	Limited number of patients; varying results; high costs
	PRP therapy	Used to enhance healing in chronic wounds	Demonstrates clinical efficacy	Variable results depending on preparation methods
	Growth factor therapy	Used in combination with other therapies to treat alopecia, burns, and other conditions	Demonstrates clinical efficacy	Need for optimization and standardized procedures
	Gene therapy (limited)	Gene therapy (CRISPR) shows promise for genetic skin disorders	Potential for long-term solutions	Ethical concerns; potential off-target effects; regulatory challenges
	Dermal fillers	Used to treat wrinkles and other cosmetic concerns	Widely used and generally considered safe	Limited duration of effects, discomfort during injections, challenges in precise delivery

Table 2 was designed in order to compare various techniques used in skin regeneration, categorized into cell-based and cell-free methods, providing a concise overview of advantages and disadvantages. The cell-based methods include cell therapy, platelet-rich plasma, and growth factors and cytokines, while the cell-free methods encompass exosomes, wound dressings, and gene therapy. Additionally, there is a section for bioengineered skin including biomaterials and nanodevices.

**TABLE 2:** Comparison of cell-based and cell-free approaches for skin regeneration.

	Technique	Advantages	Disadvantages/Limitations	References
Cell-based	Cell therapy	Differentiation potential, stimulates angiogenesis, releases growth factors, and provides immunomodulation	High cost, potential immune rejection, and decreased viability with aging	Jo et al. (2021) Lukomskyj et al. (2022) Wu et al. (2024)
	Platelet-rich plasma	Cost-effective, promotes wound healing via growth factors, easily prepared, and safe with minimal side effects	Variable results, lack of standardization, and requires further research to optimize application in specific conditions	Xu et al., 2020 Cecerska-Heryc et al. (2022) Everts et al. (2024)
Cell-free	Growth factors and cytokines	Accelerates wound healing (EGF and FGF), enhances collagen production (TGF-β), angiogenesis (VEGF), antiinflammatory effects (IL-10), potential for scar reduction	Cost and availability, short halflife, risk of overstimulation, inflammation and immune response, tumorigenic potential, unpredictable Response	Mao and Mooney (2015) Sampogna et al. (2015) Lou (2021) Colon et al., 2023
	Exosomes	Non-immunogenic, accelerates healing, promotes collagen synthesis, and poses a lower risk of immune rejection or tumorigenicity	Difficult isolation and standardization; challenges in ensuring purity and long-term stability	Wang et al. (2019) Wong et al., 2020 Zhou et al., 2023
	Wound dressings	Provides a moist environment for healing, can be infused with bioactive compounds like growth factors, and reduces pain and healing time	High production costs, complex manufacturing, and limited large scale clinical validation	Frykberg and Banks (2015) Chiu et al. (2023)
	Gene therapy (CRISPR, viral delivery)	Targets specific genetic mutations, promising for diseases like epidermolysis bullosa, can correct genetic skin conditions at the DNA level	Ethical concerns, potential off target effects, immune reactions, and regulatory challenges	Abdelnour et al. (2021) Nqoro et al. (2022) Bischof et al., 2024
Tissue engineering	Biomaterials (e.g., collagen, alginate)	Biocompatibility, promotes cell adhesion and migration, and mimics the extracellular matrix, useful in scaffolds and wound dressings	Some materials, like alginate, have low stability, and others, such as spider silk, are difficult to produce in large quantities	Ghomi et al. (2021) Surowiecka et al. (2022) Farshidfar et al., 2023
	Nanodevices (e.g., nanofiber, hydrogels, nanocrystal and nanoparticle)	Direct stimulation of cell regrowth; Antibacterial activity; Drug delivery	It is necessary to carry out nanotoxicity testing	Salehi et al., 2020; Bellu et al., 2021 Yazdi et al. (2022)
	Bioengineered skin	Offers improved healing and aesthetic outcomes for burns and wounds, reduces scarring, and restores functionality (e.g., elasticity, sensitivity)	High cost, immune rejection risks, and limitations in large scale production or tailored designs for individual patients	Boyce and Lalley (2018) Monavarian et al. (2019) Chogan et al. (2023)
	3D bioprinting	Customizable, precise production of skin tissue layers, enables faster manufacturing and reduced costs, can incorporate cells like fibroblasts	Technical limitations, including low resolution and difficulty in controlling cell distribution and viability	Pasierb et al., 2022 Wang et al., 2021a Zhang et al., 2023 Zieliński et al. (2023)

Basically, cell-based therapies rely on the biological activity of living cells (e.g., growth factors, immunomodulation), while cell-free methods utilize components derived from cells or synthetic materials to stimulate tissue repair. In general, cell-based therapies offer potential for multifaceted benefits, nevertheless they are often more complex and costly to produce than cell-free options. Also, it is possible to assume that while some cell-based therapies (like PRP) have gained clinical traction, others are still in earlier stages of development. Similarly, the table indicates cell-free options like dermal fillers are established clinically whereas gene therapy, for example, remains limited due to distinct factors such as cost, scalability, and ethical issues. In addition, the comparison sheds light on the potential safety concerns associated with each technique. While cell-based methods carry risks such as immune rejection, cell-free methods have limitations regarding long-term stability and potential off-target effects (e.g., gene therapy). This is vital for evaluating the risk-benefit profile of each approach. By highlighting the limitations of current technologies, the comparison stimulates innovation and the development of new techniques. For instance, challenges in exosome isolation and standardization may boost research into improved purification and delivery methods.

In summary, comparing cell-based and cell-free methods provides a framework for assessing the strengths and weaknesses of different regenerative approaches, contributing to decisions about research direction, clinical translation, and resource allocation within the field of skin regeneration. Addressing these limitations requires continued research, development of standardized protocols, improved manufacturing processes, robust clinical trials, and careful ethical consideration of the technologies making them more effective, affordable, and accessible.

## 7 Perspectives and future directions

Since the cave age, man has been healing his wounds, treating burns and preventing bleeding. Nowadays, the importance of aesthetics and the growth of the geriatric population is propelling the demand for skin regeneration and rejuvenation products and services. In this context, the interest in maintaining a skin with youthful appearance, the demand for treatment of disorders/disease and superficial or full-thickness skin injuries, has led to the development of regenerative medicine-based approaches, with the aim of repair, replace, regenerate, and rejuvenate (the four “R”s) the skin. In recent years we have seen rapid growth in the field of regenerative medicine-based approaches for skin.

The global regenerative medicine market size was valued at USD 29.42 billion in 2023 and it is predicted to be worth around USD 154.05 billion by 2033 with a remarkable CAGR of 18% from 2024 to 2033. The dermatology segment was valued at USD 1.87 billion in 2023 and it is expected to reach around USD 7.66 billion by 2030 (https://www.precedenceresearch.com/regenerative-medicine-market). The global scar treatment market size was estimated at USD 24 billion in 2022 and is expected to hit around USD 64.26 billion by 2032, poised to grow at a CAGR of 10.4% during the forecast period from 2023 to 2032. The growing concerns among the population regarding their aesthetic appearances are a major factor driving the growth of the global scar treatment market. Scars due to accidents and other reasons are propelling the demand for scar treatment products and services (https://www.precedenceresearch.com/scar-treatment-market).

The companies focusing on research and development are expected to lead the global regenerative medicine market (https://www.precedenceresearch.com/scar-treatment-market). Leading competitors contending in global regenerative medicine market are as follows: Integra LifeSciences Corporation (https://www.integralife.com/); Aspect Biosystems (https://www.aspectbiosystems.com/); Amgen, Inc (https://www.amgen.com/); Medtronic plc (https://www.medtronic.com/br-pt/index.html); AstraZeneca (https://www.astrazeneca.com.br/); Novartis AG (https://www.novartis.com/); Smith & Nephew plc (https://www.smith-nephew.com/pt-br); MiMedx Group (https://www.mimedx.com/); Shenzhen SibionoGeneTech Co., Ltd. (http://www.sibiono.com/en/index.aspx); Baxter (https://www.baxter.com.br/pt-br).

The innovative field of skin regeneration and rejuvenation most advances include, but are not limited to (Boyce and Lalley, 2018):

a) complete restoration of skin anatomy and physiology, anatomic and physiologic properties that may be missing by providing restoration of skin pigmentation, epidermal appendages (hair, sebaceous and sweat glands), sensory and motor innervation, vascular plexus, and subcutaneous tissues;
b) development of gene therapies for specific applications and cost-effective therapies to patients, useful to the treatment of diseases, with focus on incidence of injuries and growth of the geriatric population;
c) automated and robotic fabrication of engineered tissues to increase efficiencies and reduce costs;
d) development of instruments and medical devices for tissue engineering and regenerative medicine, organ regeneration, and organ transplantation;
e) genetic modification of autologous cells, adoption of novel stem cells-based technologies, using umbilical cord blood stem cell (allogeneic and autologous stem cells), adult stem cells (ASC), embryonic stem cells or stem cells obtained from other species (xenogeneic cell), facing and overcoming the ethical challenges due to their source of collection;
f) quantification of wounds with non-invasive biophysical instruments;
g) development of biomaterials (natural or synthetic) or biodegradable formulations that do not affect the gene expression profile of the cells through stimulation or inhibition of cell surface receptors and control of specific intracellular pathways;
h) screening of the Skin-Regenerative Potential of Peptides (Alencar-Silva et al., 2024);
i) development of 3D bioprinting techniques to biofabrication of skin tissue with its appendages, is very important due to their intrinsic capacity for automation, accuracy, reproducibility, scalability, and personalization of matrices with structural complexity (Hosseini et al., 2022);
j) the potential use of 3-dimensional organoid or stem cell-derived organoid may deliver greater patient benefits than other regenerative medicine approaches but raises new health and ethical risks (Harris et al., 2022).

## 8 Innovation and patents in skin regeneration and rejuvenation

A comprehensive analysis of the current landscape of patent applications within the field of regenerative medicine, with a particular emphasis on skin regeneration and rejuvenation has also been performed. A detailed examination of data obtained from a Google Patents search for the period 2022–2024 reveals substantial ongoing innovation and a considerable level of interest in this rapidly evolving area. Using keywords “regenerative skin”, “regenerative medicine”, “artificial skin”, “skin rejuvenation,” and “skin regeneration” yielded 22541, 67589, 55411,419 and 1,681 results, respectively.

Numerous patents cover anti-aging and anti-wrinkle cosmetics, rejuvenation methods, and the use of exosomes (from various sources), proteins (recombinant and non-recombinant), plant extracts, growth factors, and stem cells. Patents also target the development of devices and instruments for skin treatment and product application aimed at promoting regeneration. Specific applications include treatments for wound healing, chronic wounds, burns, aging, and skin diseases. However, the field of skin regeneration and rejuvenation is experiencing rapid growth in terms of patent activity and a large number of patents, significant research gaps exist, particularly in addressing the effects of ionizing radiation, neuron injury repair, and the development of functional artificial blood vessels.

Specifically, Hinsenkamp et al. (2022) reviewed tissue engineering (TE) and regenerative medicine (RM) patents from 2010 to 2020, identifying promising areas and highlighting challenges in translating research to commercial products. While many patents exist, the path to market is complex and lengthy, with regulatory hurdles and significant financial investment required.

Several patents show promise, including1) scaffold-related inventions such as the Electrospinning Company’s multiwell plate, facilitating 3D cell culture research, which is already commercially available; methodological patents focusing on cell isolation and bioprinting techniques; 3) compositional patents involving novel bioinks and materials for 3D bioprinting, particularly those that are already part of commercially available products.

The review emphasizes that, while many patents exist, most are still in early development stages. Only a small fraction has reached commercialization, highlighting the significant challenges in this field. The authors emphasize the lengthy timelines, regulatory complexities, and funding challenges involved in moving promising TE and RM technologies from the laboratory to the market. Further innovation and research are needed to overcome these limitations.

## 9 Regulatory and ethical

The regenerative medicine-based therapies for different pathologies, including those of the skin, are generally considered safe, although there is still a need to overcome some limitations, especially those related to tissue origin and donor factors, discrepancies in isolation and culture procedures, risk of adverse effects, such as tumorigenicity, and some ethical regulatory restrictions (Shimizu et al., 2022). Safety and efficacy of skin substitutes are regulated in the USA by the US Food and Drug Administration (FDA), which released a guidance document “Regulatory Considerations for Human Cells, Tissues, and Cellular and Tissue-Based Products (HCT/Ps): Minimal Manipulation and Homologous Use” (https://www.fda.gov) and International Society of Stem Cell Research (ISSCR) communications (https://www.isscr.org/policy). The ASTM (American Society for Testing and Materials, https://www.astm.org) F2311-08 Standard Guide for Classification of Therapeutic Skin Substitutes (defines terminology and provides classification by clinical use for products that can be substituted for tissue grafts of human or animal tissue in medical and surgical therapies of skin lesions) and F3163-16, Standard Guide for Classification of Cellular and/or Tissue-Based Products for Skin Wounds (CTPs) (defines terminology for description of CTPs for skin wounds), are used by wound care (1) scientists when developing new technology and submit dossier to agencies such as the FDA, (2) manufacturers when producing or launching new technology, (3) researchers when designing clinical trials, and (4) professionals to guide their clinical practice (Drueck, 2018).

Several ethical implications related to the development from bench to bedside: (1) animal experimentation, (2) handling human tissue, (3) informed consent, (4) therapeutic potential, (5) risk and safety, (6) clinical translation, and (7) societal impact. And three themes represent ethical safeguards relevant to all developmental phases: (8) scientific integrity, (9) regulation, and (10) patient and public involvement need more ethical and social reflection (Kanter et al., 2023).

## 10 Conclusion

Regenerative therapies hold great promise in medicine, but their effectiveness can vary significantly between individuals. Factors such as disease severity, genetic background, and overall health influence the outcomes of these treatments. Additionally, many regenerative therapies remain in experimental or early clinical stages, often making them expensive and limiting accessibility for patients. Beyond cost, there are substantial regulatory hurdles, as these treatments require thorough clinical testing and approval to ensure safety and efficacy, a process that is often lengthy and complex. Furthermore, technical challenges abound in the production and application of regenerative materials like stem cells and engineered tissues, requiring specialized expertise and equipment that not all medical centers can provide. Innovative research is focused on developing minimally invasive aesthetic treatments and advancing biological skin substitutes. These innovations aim to reduce morbidity from both acquired and congenital skin diseases, as well as replace or regenerate soft tissues lost or damaged due to aging, injuries, scars, or wrinkles. The ultimate goal is to restore a rejuvenated, natural, and aesthetically pleasing appearance to the skin. Despite the promising potential of regenerative medicine, existing regulations and guidelines are insufficient to address the associated risks. The interdisciplinary nature of this field, encompassing stem cell biology, biomaterial science, gene therapy, tissue engineering, and more, needs a thorough ethical and social reflection to ensure responsible development.
